# Poor or fair self-rated health is associated with depressive symptoms and impaired perceived physical health: A cross-sectional study in a primary care population at risk for type 2 diabetes and cardiovascular disease

**DOI:** 10.1080/13814788.2019.1635114

**Published:** 2019-07-08

**Authors:** Ansa Talvikki Rantanen, Jyrki Jaakko Antero Korkeila, Hannu Kautiainen, Päivi Elina Korhonen

**Affiliations:** a Department of General Practice, University of Turku and Turku University Hospital, Turku, Finland;; b Salo Health Center, Salo, Finland;; c Department of Psychiatry, University of Turku and Turku University Hospital, Turku, Finland;; d Department of Psychiatry, Hospital District of Satakunta, Pori, Finland;; e Folkhälsan Research Center, Helsinki, Finland;; f Unit of Primary Health Care, Kuopio University Hospital, Kuopio, Finland;; g Central Satakunta Health Federation of Municipalities, Harjavalta, Finland

**Keywords:** Heart and circulation, anxiety/depression/somatization/surmenage/sleep, general practice/family medicine, general, cross-sectional designs

## Abstract

**Background:** Psychosocial factors such as depressive symptoms should be considered when assessing cardiovascular (CV) risk. Depressive symptoms are suggested to be associated with poor perception of one’s health, i.e. self-rated health (SRH). Thus, assessing SRH could be a practical tool in CV risk prediction. However, SRH may also emphasize physical, mental or social aspects.

**Objectives:** To assess the relationship of SRH and depressive symptoms, classic CV risk factors and perceived physical health among persons at risk for type 2 diabetes (T2D) and cardiovascular disease (CVD).

**Methods:** In this cross-sectional study in a primary care population, 2555 persons (mean age 58 ± 7, 56% women) at risk for T2D or CVD were evaluated. Generalized linear statistical models were used to evaluate the association of depressive symptoms (Beck’s Depression Inventory score ≥10), CV risk factors, and perception of SRH and physical health (assessed by Short Form Health Survey).

**Results:** Poor or fair health was reported by 40% of the participants. They had more unhealthy lifestyle habits and CV risk factors than subjects rating their health as at least good. Among those with poor or fair SRH, the prevalence of depressive symptoms was 36% and associated with perception of physical health.

**Conclusion:** Poor SRH is associated with depressive symptoms and impaired perceived physical health. Assessing SRH might be useful for detecting possible depressive symptoms in patients in CV risk management and diabetes care.

KEY MESSAGES

- Poor or fair self-rated health associates with depressive symptoms and cardiovascular risk factors
- Depressive symptoms associate with impaired subjective perception of physical health among those with poor or fair self-rated health
- Assessing self-rated health might be a practical tool in cardiovascular risk prevention activities in primary care

## Introduction

Psychosocial factors including depressive symptoms should be considered as risk modifiers in cardiovascular (CV) risk prediction but it has been suggested that detection of depressive symptoms by general practitioners is inadequate [1–3]. Poor perception of one’s health, i.e. self-rated health (SRH), is strongly associated with depressive symptoms [4]. Furthermore, poor SRH has been previously associated with increased risk for undetected hypertension, diabetes, renal insufficiency and peripheral arterial disease [5], and has even been shown to predict CV events [6]. Thus, assessing SRH might be an efficient way to detect depressive symptoms and find those at particular CV risk. However, for some individuals health equals a lack of physical diseases or disability, whereas others may emphasize mental or social aspects. The present study aimed to assess the relationship of SRH and depressive symptoms, classic CV risk factors and perceived physical health among middle-aged subjects at risk for type 2 diabetes (T2D) or cardiovascular disease (CVD). We hypothesized that poor SRH associates with depressive symptoms but perceived physical health might modify the association.

## Methods

The present study is a cross-sectional analysis of the association between SRH, depressive symptoms and perceived physical health in a population of persons at risk for T2D or CVD.

### Ethical approval

The ethics committee of the Satakunta hospital district reviewed and approved the study protocol and consent forms on 3 October 2004. All participants provided written informed consent for the project and subsequent medical research.

### Study population

**Figure 1.**
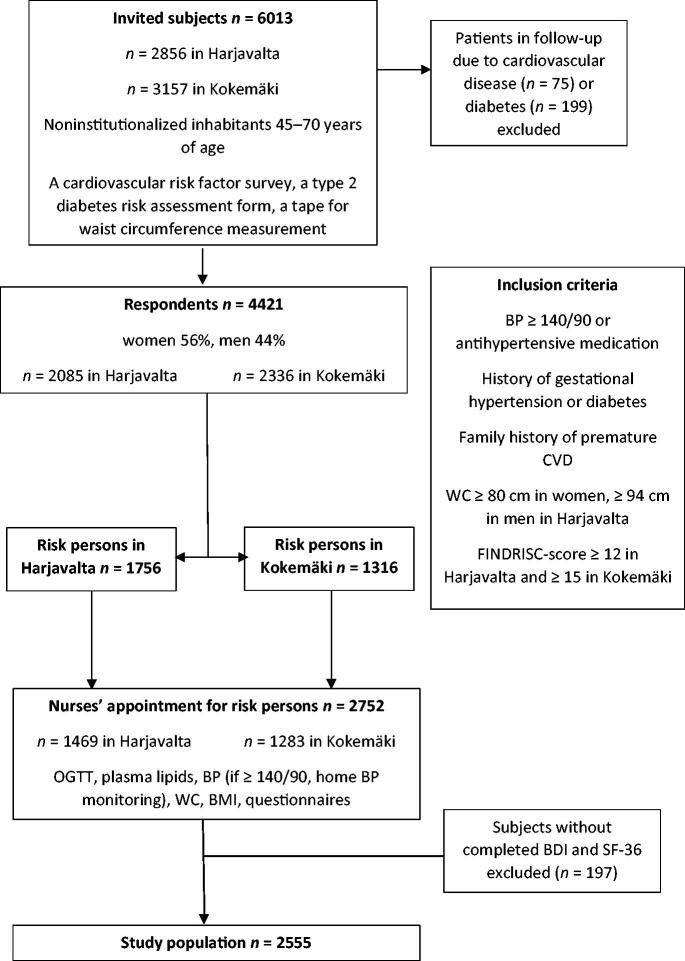
Flowchart of the study design. BP: blood pressure; CVD: cardiovascular disease; WC: waist circumference; OGTT: oral glucose tolerance test; BMI: body mass index; BDI: Beck’s Depression Inventory; SF-36: Short Form Health Survey.

A population survey, the Harmonica Project (Harjavalta Risk Monitoring for Cardiovascular Disease) [7], was conducted in the towns of Harjavalta and Kokemäki from August 2005 to September 2007. A cardiovascular risk factor survey, a tape for waist circumference measurement, and a T2D risk assessment form (FINDRISC, Finnish Diabetes Risk Score, available from www.diabetes.fi/ English) were mailed to all noninstitutionalized inhabitants aged 45–70 years (*n* = 6013) [8]. The response rate was 74%. Inclusion criteria were the latest blood pressure (BP) measurement ≥140/90 mmHg, use of antihypertensive medication, history of gestational diabetes or hypertension, family history of premature CVD, waist circumference at the level of the navel ≥80 cm in women and ≥94 cm in men in Harjavalta, and FINDRISC-score ≥12 points in Harjavalta (an estimated 1 in 6 will develop T2D within 10 years) or ≥15 points in Kokemäki (an estimated 1 in 3 will develop T2D within 10 years) [8]. Different FINDRISC-scores were used for logistic reasons. Exclusion criteria were established CVD, renal disease or diabetes. The inclusion criteria were met by 3072 subjects, of whom 2752 attended a study nurses’ appointment. For the analysis described here, only subjects with completed Beck’s Depression Inventory (BDI) and Short Form Health Survey (SF-36) were included (*n* = 2555) [9,10]. The study design and formation of the study population are illustrated in Figure 1.

### Measurements

The study subjects completed self-administrated questionnaires and attended an enrolment examination performed by a trained nurse. The questionnaires measured education (upper secondary school completed/not), cohabiting (yes/no), current smoking (yes/no), alcohol consumption (Alcohol Use Disorders Identification Test, AUDIT [11]; AUDIT score ≥8 as a cut-off for harmful alcohol use) and leisure-time physical activity (LTPA) level (classified as high: LTPA for at least 30 min at a time for six or more times a week; moderate: LTPA for at least 30 min at a time for four to five times a week; low: LTPA for at least 30 min at a time for a maximum of three times a week). Depressive symptoms were assessed by BDI, version BDI-21; BDI scores ≥10 indicating depressive symptoms and health-related quality of life by the Short Form Health Survey (SF-36), version 1.0, available and validated in multiple languages [10,12]. The first question of the SF-36, ‘In general, how would you rate your health?’ assesses SRH from 1 = poor to 5 = excellent. For the present study, SRH was categorized into three levels as follows: level I: poor or fair; level II: good; level III: very good or excellent. The SF-36 physical component summary score assessed the study subjects’ perception of their physical health.

BP was measured with subjects in a sitting posture, after resting for at least five minutes with the cuff placed on the arm. Definition of hypertension was BP ≥140/90 mmHg or use of antihypertensive medication. Body mass index (BMI) was calculated as weight (kg) divided by the square of height (m^2^). Obesity was defined as BMI ≥30.0 kg/m^2^.

Laboratory tests (fasting glucose, two-hour glucose tolerance test, total cholesterol) were determined in blood samples obtained after at least 12 h of fasting. Glucose metabolism disorders were categorized into T2D (fasting glucose ≥7.0 mmol/l or two-hour post-load plasma glucose ≥12.2 mmol/l) and intermediate hyperglycaemia (fasting glucose 6.1–6.9 mmol/l or two-hour plasma glucose 8.9–12.1 mmol/l) [13].

Data on regularly used medication was gathered from the questionnaire and medical records.

### Statistical analysis

The descriptive statistics were presented as means with SDs or as counts with percentages. Statistical significances for the unadjusted hypothesis of linearity across the categories of SRH were evaluated by using the Cochran–Armitage test for trend and analysis of variance with appropriate contrast.

The adjusted hypothesis of linearity (orthogonal polynomial) of SRH and depressive symptoms was evaluated using generalized linear statistical models (e.g. analysis of covariance and logistic models) with appropriate distribution and link function. Models included age, cohabiting status, smoking, LTPA, BMI, years of education, plasma glucose level, systolic BP level, and medication as covariates. The Shapiro–Wilk W-test evaluated the normality of variables. All statistical analyses were carried out with Stata, version 15.1 (StataCorp, College Station, TX, USA).

## Results

### Characteristics of the study population

We evaluated 2555 subjects (mean age 58 ± 7 years, 56% women) at increased risk for T2D and CVD, but without manifested CVD, diabetes or renal disease. The characteristics of the study population according to the categories of SRH are presented in Table 1. Poor or fair health was reported by 40%, good health by 31%, and very good or excellent health by 30% of the subjects. SRH was negatively associated with BDI parameters and medication for depression/anxiety, and the co-factors of age, current smoking, low LTPA, systolic BP, hypertension, glucose parameters, BMI parameters, and medication for hypertension and lipid disorders. SRH was positively associated with the co-factors of education, cohabiting, and high LTPA.

**Table 1.** Characteristics of the subjects according to self-rated health.

		Self-rated health			
	All *n* = 2555	Level I *n* = 1013	Level II *n* = 781	Level III *n* = 761	*P*-value*
Age, mean (SD)	58 (7)	60 (7)	57 (7)	57 (7)	<0.001
Females, *n* (%)	1420 (56)	560 (55)	450 (58)	410 (54)	0.63
Education years, mean (SD)	10.4 (2.7)	9.7 (2.5)	10.7 (2.8)	11.0 (2.8)	<0.001
Cohabiting, *n* (%)	1994 (78)	770 (76)	606 (78)	618 (81)	0.01
Current smoking, *n* (%)	446 (18)	199 (20)	126 (16)	121 (16)	0.035
AUDIT score, mean (SD)	4.7 (4.9)	4.7 (5.4)	4.7 (4.7)	4.5 (4.2)	0.28
AUDIT score ≥ 8, *n* (%)	516 (20)	215 (21)	163 (21)	138 (18)	0.12
LTPA, *n* (%)					<0.001
Low	460 (18)	230 (23)	133 (17)	97 (13)	
Moderate	1284 (50)	499 (49)	394 (51)	391 (51)	
High	805 (32)	281 (28)	252 (32)	272 (36)	
Blood pressure, mmHg, mean (SD)					
Systolic	140 (19)	142 (18)	140 (19)	139 (18)	<0.001
Diastolic	84 (10)	84 (10)	84 (10)	84 (10)	0.77
Hypertension (BP ≥ 140/90), *n* (%)	1352 (53)	571(56)	405(52)	376 (50)	0.004
Total cholesterol, mmol/l, mean (SD)	5.4 (1.0)	5.4 (1.0)	5.4 (1.0)	5.4 (0.9)	0.89
Total cholesterol ≥ 5.0, *n* (%)	1681 (66)	656 (65)	519 (67)	506 (67)	0.41
Plasma glucose, mmol/l, mean (SD)					
Fasting	5.6 (1.2)	5.7 (1.3)	5.6 (1.1)	5.5 (0.9)	<0.001
Two-hours post-load	7.4 (2.3)	7.8 (2.5)	7.3 (2.1)	7.1 (2.1)	<0.001
Glucose disorder, *n* (%)					
Intermediate hyperglycaemia	605 (24)	290 (29)	171 (22)	144 (19)	<0.001
New diagnosis of type 2 diabetes	152 (6)	81 (8)	43 (6)	28 (4)	<0.001
Body mass index, kg/m^2^, mean (SD)	28.8 (5.0)	29.9 (5.4)	28.4 (4.7)	27.7 (4.3)	<0.001
Body mass index ≥ 30.0 kg/m^2^, *n* (%)	832 (33)	428 (42)	216 (28)	188 (25)	<0.001
BDI score, mean (SD)	6.0 (5.6)	8.7 (6.5)	5.0 (4.5)	3.5 (3.7)	<0.001
BDI score ≥ 10, *n* (%)	511 (20)	363 (36)	102 (13)	46 (6)	<0.001
Medication, *n* (%)					
Blood pressure	957 (37)	569 (46)	226 (29)	162 (21)	<0.001
Lipid disorders	325 (13)	172 (17)	89 (11)	64 (8)	<0.001
Depression/anxiety	104 (4)	74 (7)	17 (2)	13 (2)	<0.001

* *P*-value of linearity (linear trend) across categories of SRH level.

Level I: poor or fair; Level II: good; Level III: very good or excellent; AUDIT: Alcohol Use Disorders Identification test; LTPA: leisure-time physical activity; BP: blood pressure; BDI: Beck’s Depression Inventory.

### Association of depressive symptoms, perception of physical health and SRH

**Figure 2.**
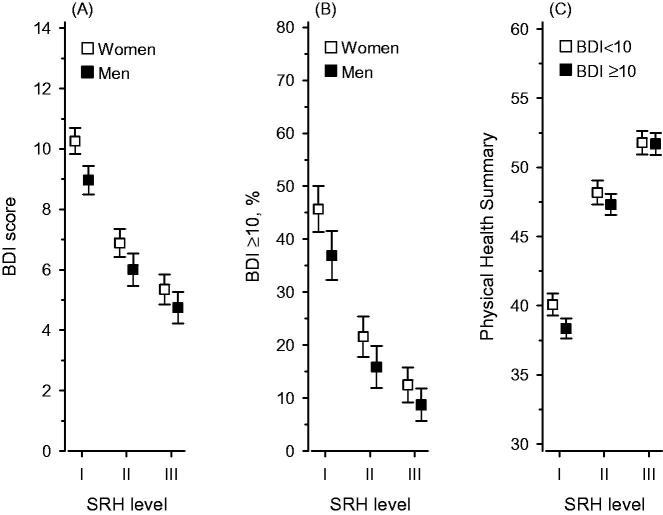
Association of BDI mean score (A), depressive symptoms (BDI ≥ 10) (B), physical health summary score (C) and self-rated health, adjusted for age, cohabiting status, smoking, leisure-time physical activity, body mass index, education, systolic blood pressure level, plasma glucose level, and medication usage; (C) is also adjusted for gender. Whiskers show 95% confidence intervals. BDI: Beck’s Depression Inventory; SRH: self-rated health level; I: poor or fair; II: good; III: very good or excellent.

Figure 2 shows the relationship between categories of SRH and BDI mean score (A), dichotomized prevalence of depressive symptoms (B), and physical health summary score (C), respectively. A linear decrease in the mean score of BDI and the prevalence of depressive symptoms (defined as BDI ≥10) was associated with better SRH (*P* <0.001). Women had more depressive symptoms than men in all SRH categories (*P* <0.001). The interaction between the presence of depressive symptoms and SRH was not significant (*P* = 0.98). The physical health summary score was higher with increasing levels of SRH (*P* <0.001), but there was an interaction between the presence of depressive symptoms and physical health on SRH (*P* = 0.009). Among persons with poor or fair SRH, the presence of depressive symptoms modified their perception of their physical health (*P* <0.001).

## Discussion

### Main findings

Among an apparently healthy, middle-aged primary care population at risk for T2D and CVD, SRH seems to have an inverse relationship with depressive symptoms, and a positive relationship with physical health. However, depressive symptoms seem to modify the subject’s perception of his or her physical health among those experiencing poor or fair health. Subjects considering their health poor or fair more often had an adverse lifestyle and CV risk factors (specifically obesity, hypertension and glucose disorders) compared to those with better ratings of their perceived health. Moreover, 45% of women and 35% of men with poor or fair SRH had depressive symptoms.

### Strengths and limitations

The strengths of our study are that the data comes from a community-based representative sample of subjects at risk for T2D and CVD, bias regarding chronic illnesses was excluded, trained medical staff made clinical measurements, and the SF-36 questionnaires were filled in before the subjects attended nurses’ appointments. The study also has limitations: the cross-sectional design prevents us to draw conclusions regarding the causal association of depressive symptoms and SRH, self-reporting of physical activity, smoking and alcohol usage may be unreliable. Our data was gathered in 2005–2007 but the prevalence of many CV risk factors, especially obesity, has since then only increased in Europe [14].

### Interpretation of the results in relation to existing literature

Our results indicate that a subject’s self-rating of poor or fair health is associated with depressive symptoms. Thus, by using a few seconds asking the patient’s perception of his or her overall health, primary care practitioners and nurses may enhance the detection of depressive symptoms and recognition of depression, which has been found to be poor [2], and often delayed [3]. We suggest that SRH assessment could be a practical tool to consider psychological risk factors in CV risk management and diabetes care.

The novel finding in our study is that among subjects experiencing poor or fair health, the perception of physical health was lower in depressive than in non-depressive persons. On the contrary, depressive symptoms had no impact on the perception of physical health of persons with good SRH. Among those with poor or fair SRH, depressive symptoms may affect the adoption of a healthy lifestyle, and treatment of depression would be worth prioritizing before attempting to make changes in lifestyle.

### Implications for further research

In the future, longitudinal studies on incorporating SRH assessment into CV risk management and diabetes care are needed to determine whether assessing SRH promotes detection and treatment of depressive symptoms.

## Conclusion

Among subjects at risk for T2D or CVD, SRH is associated with depressive symptoms and perception of physical health. Subjects with poor or fair SRH and depressive symptoms also have a poor perception of physical health.
